# The status of drowning in 1 to 5-month-old children outside the hospital, Tabriz between 2013 and 2017 

**Published:** 2019-07

**Authors:** Masoumeh Ansari, Ahmad Mardi, Naser Rahbari-Farzoo

**Affiliations:** ^*a*^Deputy of Health, Tabriz University of Medical Sciences, Tabriz, Iran.; ^*b*^Deputy of Administrative Affairs of Healthcare Facilities, Tabriz University of Medical Sciences, Tabriz, Iran.; ^*c*^Department of Health, Tabriz University of Medical Sciences, Tabriz, Iran.

## Abstract

**Background:**

Out of all types of accidents, falling into water, immersion and the resulting drowning, are among the unintentional accidents in children which can be prevented. The purpose of this study was to investigate the status of drowning of children aged 1 to 59 months outside hospital in Tabriz from 2013 to 2017.

**Methods:**

In this descriptive cross-sectional study, the mortality data of children and referrals from suburban cities affiliated to Tabriz University of Medical Sciences were investigated.

**Results:**

Of 14 reported deaths in children due to drowning, three cases (21%) were drowned in the swimming pool/traditional Iranian pool (howz), four cases (29%) due to flood, and seven cases (50%) in natural water resources and rivers. Sixty percent of deaths had occurred in rural and 40% in urban areas. The most common age was 12 months to 4 years old.

**Conclusions:**

Since children under the age of 5 and especially between the ages of 1-4 years old were the groups with the highest risk of drowning, this age group should be focused for prevention strategies, by fencing the pools, preventing children from playing under the bridges and on the way of flood, and not leaving them alone.

**Keywords:**

Drowning, Tabriz, Children aged 1-59 months

